# Arterial Hypertension—Oxidative Stress and Inflammation

**DOI:** 10.3390/antiox11010172

**Published:** 2022-01-17

**Authors:** Julia Krzemińska, Magdalena Wronka, Ewelina Młynarska, Beata Franczyk, Jacek Rysz

**Affiliations:** Department of Nephrology, Hypertension and Family Medicine, Medical University of Lodz, ul. Zeromskiego 113, 90-549 Lodz, Poland; julia.krzeminska@stud.umed.lodz.pl (J.K.); magdalena.wronka@stud.umed.lodz.pl (M.W.); bfranczyk-skora@wp.pl (B.F.); jacek.rysz@umed.lodz.pl (J.R.)

**Keywords:** arterial hypertension (AH), blood pressure (BP), oxidative stress, inflammation, reactive oxygen species (ROS), hypertension therapy

## Abstract

Arterial hypertension (AH) is a major cause of cardiovascular diseases (CVD), leading to dysfunction of many organs, including the heart, blood vessels and kidneys. AH is a multifactorial disease. It has been suggested that the development of each factor is influenced by oxidative stress, which is characterized by a disturbed oxidant-antioxidant balance. Excessive production of reactive oxygen species (ROS) and an impaired antioxidant system promote the development of endothelial dysfunction (ED), inflammation and increased vascular contractility, resulting in remodeling of cardiovascular (CV) tissue. The hope for restoring the proper functioning of the vessels is placed on antioxidants, and pharmacological strategies are still being sought to reverse the harmful effects of free radicals. In our review, we focused on the correlation of AH with oxidative stress and inflammation, which are influenced by many factors, such as diet, supplementation and pharmacotherapy. Studies show that the addition of a single dietary component may have a beneficial effect on blood pressure (BP) values; however, the relationship between the antioxidant/anti-inflammatory properties of individual dietary components and the hypotensive effect is not clear. Moreover, AH pharmacotherapy alleviates the increased oxidative stress, which may help prevent organ damage.

## 1. Introduction

Arterial hypertension (AH) is a chronic condition characterized by a blood pressure (BP) value at which the benefits of therapy outweigh its risks. According to ESC/ESH guidelines, AH is defined as systolic blood pressure (SBP) ≥ 140 mm Hg and/or diastolic blood pressure (DBP) ≥ 90 mm Hg [1]. However, the ACC/AHA hypertension guidelines state that AH should be diagnosed at systolic and diastolic BP values of ≥130 and/or ≥80 mm Hg, respectively [2]. The diagnosis of AH should be based on repeated office BP measurements or BP measurements using ambulatory blood pressure monitoring (ABPM) and/or home blood pressure monitoring (HBPM) [1]. It is estimated that 1.13 billion people suffer from AH worldwide, and that this number will increase by approximately 15–20% by 2025 [1]. Most cases (90%) of AH are classified as essential, or primary. Secondary AH is when the etiology is known, and occurs in only about 10% of diagnosed AH [3]. AH may be caused by kidney, cardiovascular (CV), neurological or endocrine diseases, such as sleep apnea, hyperaldosteronism, renal parenchymal diseases, renal artery stenosis, thyroid diseases, Cushing syndrome or pheochromocytoma [3,4].

The treatment of AH is based on two fundamental pillars: lifestyle modification and pharmacotherapy [1,4], whose effects on oxidative stress and inflammation have been described in this review. The primary goal of therapy for all patients has been defined as a BP < 140/90 mm Hg. With good treatment tolerance, the goal should be ≤130/80 mm Hg. Lifestyle changes aimed at lowering BP should be based on a balanced, low-salt diet, increased fruit and vegetable intake, a reduction in alcohol consumption and adequate potassium intake. Smoking cessation, maintaining an appropriate body weight and regular physical activity are also recommended [1,4].

In many patients it is necessary to implement pharmacological treatment of AH. Pharmacotherapy is based on five classes of drugs and their combinations: angiotensin converting enzyme inhibitors (ACE-I), angiotensin receptor blockers (ARBs), beta-blockers, calcium channel blockers (CCBs), thiazides and thiazide-like diuretics. The therapy may be supplemented with categories of drugs such as alpha-blockers, centrally acting drugs and mineralocorticoid receptor antagonists [1]. However, it is worth noting that according to the Prospective Urban Rural Epidemiology (PURE) study, only 46.5% of patients with AH are aware of the diagnosis, and only 32.5% of those treated have their blood pressure controlled [5]. AH is an important cause of cardiovascular diseases (CVD) and death worldwide [5]. It also leads to dysfunction of many organs, including, but not limited to, the heart and blood vessels, kidneys, eyes (hypertensive retinopathy) and increased risk of brain damage [1].

AH is a disease in which incidence increases with age [6]. It is a multifactorial illness, and it has been suggested that the development of each factor is influenced by oxidative stress, which is characterized by a disturbed oxidative-antioxidative balance [7,8,9]. Oxidative stress may be induced by overproduction of reactive oxygen species (ROS) or an ineffective antioxidant system [7,8]. All the factors leading to the development of AH contribute to such abnormalities as endothelial dysfunction (ED), lipid peroxidation, inflammation and increased contractility and vascular remodeling [9,10,11]. Consequently, large flexible arteries become stiff, lose flexibility and have other changes to mechanical properties that lead to a reduction in compliance, resulting in elevated SBP [6]. Moreover, renin angiotensin system (RAS) activation deepens remodeling of the CV tissue, promoting inflammation [12]. All these physiological changes appear with increasing age, but AH accelerates these processes [6].

## 2. Basic Information about Disturbed Oxidant-Antioxidant Balance

The proper functioning of the organism may be disturbed by many factors—one of them is the imbalance between the amount of oxidants and antioxidant production. The condition characterized by an excessive production of ROS is called oxidative stress, however, it may also be caused by an insufficient antioxidant system [7,8]. Increased oxidative stress leads to an intensified antioxidant defense, however, it is not sufficient to inactivate the ROS. Predominance of ROS results in DNA, protein and lipid damage, leading to cell and tissue impairment [13,14].

ROS are derivatives of molecular oxygen, and some of them, called free radicals, contain at least one unpaired electron, which contributes to their high reactivity. Among ROS, we can distinguish such reactive forms as superoxide (O_2_^•−^), hydrogen peroxide (H_2_O_2_), singlet oxygen, hydroxyl radicals, peroxyl radicals, alkoxyl radicals, peroxynitrite, hypochlorous acid and ozone. Table 1 shows examples of the most common ROS, categorized as either oxygen-free radicals or non-radical ROS [7,14]. ROS may also regulate signaling pathways by direct interplay with critical signaling molecules, contributing to signaling in such cellular processes as proliferation and survival through MAP, PI3 and/or PTEN kinases, or in the regulation of antioxidant genes through thioredoxin and/or Nrf2 [8].

The main producers of ROS in the organism are mitochondria and phagocytic cells. Mitochondria produce ROS in the mitochondrial respiratory chain, while phagocytic cells create it through the enzyme nicotinamide adenine dinucleotide phosphate oxidase (NADPH oxidase) in the process of respiratory burst. Examples of other sites where ROS may be produced endogenously include the endoplasmic reticulum (ER), peroxisomes or by the action of xanthine oxidases (XO) [7,8]. However, the external environment may also be the source of ROS, including air pollutants, radiation or xenobiotics [8,14]. For example, gamma rays from electromagnetic radiation can split water in the organism to create hydroxyl radical (^•^OH) [15].

The combination of unpaired electrons from two free radicals creates a non-radical ROS because a covalent bond is formed. However, if a free radical reaction with a non-radical is initiated, a vicious circular mechanism will arise in which newly formed radicals will promote the formation of even more new radicals. This situation occurs in the lipid peroxidation mechanism where reactive radicals attack the fatty acid side chains [15].

Antioxidants are the organism’s line of defense against the harmful effects of ROS. They are substances aiming to prevent, reduce and repair destruction caused by ROS. The main mechanisms of their action include the direct removal of the produced ROS, inhibition of ROS production in cells and repair of existing damage. There are many sources of antioxidants, some of them are produced endogenously, such as superoxide dismutase (SOD); others we can derive from food, such as vitamin C [14].

## 3. Molecular Aspects of Oxidative Stress in Arterial Hypertension

According to the mosaic theory, the development of AH is influenced by many regulatory mechanisms, such as anatomical, genetic, endocrine, humoral, hemodynamic, environmental or adaptive neural factors, and, presumably, oxidative stress may contribute to each of the factors mentioned above [9]. Physiologically, ROS as signaling molecules play an essential role in the regulation of endothelial function. However, in a situation of overproduction and overactivity, they promote ED, lipid peroxidation, inflammation, increased contractility and vascular remodeling, leading to the development and progression of AH [9,10,11].

Vascular production of ROS in people suffering from AH is characterized by levels of O_2_^•−^ and H_2_O_2_ that are significantly higher than in healthy people. Further, each ROS involved in cell signaling has different chemical properties, resulting in stimulation of separate signaling pathways [16,17]. Thus, O_2_^•−^ has a short half-life, while H_2_O_2_ has a longer half-life and the ability to diffuse over greater distances. Moreover, O_2_^•−^ contributes to the inactivation of vasodilation and, consequently, to the development of ED and vasoconstriction, while H_2_O_2_ has a vasodilating effect, including on the coronary vessels [17].

The main producers of free radicals in blood vessels are activated pro-oxidative enzymes, such as NADPH oxidase, XO and uncoupled nitric oxide (NO) synthases; however, increasing evidence is leaning towards the growing role of mitochondrial and ER enzymes [9,12,17]. Their activity is regulated by many factors, including humoral factors (e.g., cytokines) and physical factors (e.g., stretching). Additionally, the nature of blood flow through the vessels deserves special attention [11,17]: laminar flow has a protective effect on the vessels by stimulating the antioxidant defense and the production of NO, but oscillatory shear induces oxidative impairment, leading to vascular damage and inflammation [17].

The harmful effects of ROS contribute to inflammatory reactions in blood vessels through many different mechanisms, one of which is an increase in the production of proinflammatory cytokines [14]. In lipopolysaccharide-induced sepsis, mitochondrial O_2_^•−^/H_2_O_2_ has been shown to activate inflammatory cells by stimulating the expression of proinflammatory cytokines. It is worth noting the relationship between the quantity of mitochondrial reactive oxygen species (mtROS) and the increased production and release of proinflammatory cytokines from blood cells, as well as the accumulation of damaged mitochondria producing ROS in greater amounts as a result of the activation of the NLRP3 inflammasome, which is associated with the subsequent overproduction of proinflammatory cytokines. Inflammasomes in inflammatory conditions cause an increased permeability of the mitochondrial membrane to H_2_O_2_, which upon accessing the cytoplasm leads to the induction of proinflammatory pathways and activates inflammatory cells. There is speculation that endothelin-1 (ET-1) may mediate oxidative stress and inflammation by stimulating the ET-1 receptor, which promotes O_2_^•−^ formation through the induction of NADPH oxidase. Furthermore, inflammatory cells (especially T cells and monocytes/macrophages) appear to play a key role in the development of oxidative stress, ED and increased NADPH oxidase activity. Inhibition of ET-1 receptors seems to be an effective method for the suppression of inflammatory cells. It has been noted that mtROS was a potent trigger for the activation of NADPH oxidase 2 (Nox2), leading to the activation and influx of myelomonocytes, and that mitochondrial H_2_O_2_ stimulated T cells to increase tumor necrosis factor-α (TNF-α) production. ROS may also affect inflammatory processes by regulating the formation of extracellular neutrophil traps (NETs) with NADPH oxidase activity. Moreover, redox pathways may modulate transcription factors involved with inflammatory pathways, e.g., NF-κB and Nrf2, and may also reduce inflammatory mediators, e.g., DAMP and S100 [18].

**Figure 1:**
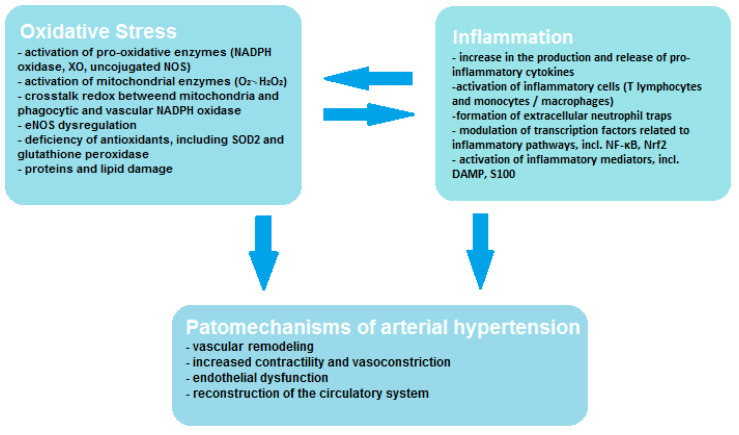
Correlation between oxidative stress, inflammation and hypertension.

Vascular endothelial cells contain smaller amounts of mitochondria; however, oxidative stress of mitochondrial origin has been shown to be related to ED [19]. Mitochondria may affect vascular function through many mechanisms, including increased ROS production in systemic and peripheral circulation and deficiency of aldehyde dehydrogenase and mitochondrial redox signaling pathways. The mitochondrion not only produces large amounts of ROS in the form of O_2_^•−^ and H_2_O_2_, but it is also very sensitive to oxidative stress, which leads to mitochondrial damage and promotes increased ROS production [9]. During oxidative phosphorylation, mitochondria produce significant amounts of ROS in the electron transport chain (ETC) and by the other oxidant enzymes that bind to the ROS systems found in other cellular organelles and function to signal inflammation or cell death. Through these processes, mitochondria are the main bridge enabling signals to be sent between ROS produced in other organelles and the DAMP detection pathways (e.g., inflammasomes), which allows metabolic homeostasis to be maintained. Moreover, it has been demonstrated that extra-mitochondrial ROS may transmit signals to the mitochondria and initiate the production of mtROS by influencing the activity of ETC enzymes either through the mitochondrial permeability transition pore (MPTP). Mitochondria may also regulate inflammatory pathways by promoting mtROS-mediated inflammation through activation of NLRP3- inflammasome/caspase-1, which contributes to cell proliferation or migration mediated by Rac1 or the inflammatory transcription factor NF-kB, and also promotes apoptosis by activating caspase-3 [20]. As can be seen, the mechanism of ROS formation and interaction is a complex process, exemplified by the existing crosstalk redox between mitochondria and phagocytic and vascular NADPH oxidase, and this interaction influences vascular function through endothelial nitric oxide synthase (eNOS) dysregulation. Crosstalk redox can be initiated by so-called kindling radicals, which are formed in the mitochondria as a result of aging or in response to nitroglycerin therapy. O_2_^•−^/H_2_O_2_ of mitochondrial origin mainly causes activation of Nox2 and increased production of cytosolic ROS by NADPH oxidase. However, crosstalk redox can result from reverse transmission, in which the phagocytic NADPH oxidase generates kindling radicals that affect the mitochondria, resulting in increased production of mtROS [21]. In angiotensin-II (AT-II)-induced hypertension, it was noticed that free radicals of mitochondrial origin also have the ability to convert xanthine dehydrogenase (XDH) into XO, which leads to decoupling of eNOS, resulting in the formation of O_2_^•−^ instead of NO, which translates into an unfavorable phenotype [22]. The mutual correlation between oxidative stress, inflammation and the pathogenesis of hypertension is shown in Figure 1. It is worth paying attention to the interplay between oxidative stress and inflammation, which activates and enhance its effects.

The development of elevated BP depends on increased ROS production, however, a deficiency of antioxidants, including SOD2 and glutathione peroxidase, also plays an important role in the pathogenesis of AH [9]. Cross-sectional analysis undertaken by Brunelli and colleagues [16] has evaluated the relationship between oxidative imbalance and established CVD risk factors, including SBP, in a healthy population. By photometric measurement the reactive oxygen metabolite (ROM) and antioxidant potential were investigated and evaluated. During the study, SBP values were analyzed and related to the antioxidant barrier efficacy and oxidation state. It was shown that with increasing values of SBP, ROM values remained constant, however, a significant reduction in antioxidant capacity was observed at and above 150 mmHg [16].

Pregnant women are likewise exposed to the adverse effects of oxidative stress in the presence of preeclampsia, which is a hypertensive disorder and a life-threatening condition for both mother and fetus. Meta-analysis undertaken by Taravati A. and Tohidi F. [23] estimated the role of oxidative stress and antioxidant response in women with preeclampsia. Free radicals and oxidative stress appear to play a substantial role in the process of preeclampsia; however, it is uncertain whether antioxidant deficiency and oxidative stress are a direct cause of this condition or just its consequence. It was observed that the level of malondialdehyde (MDA), which is a lipid peroxidation product, is visibly increased in preeclampsia compared to normal pregnancy. Moreover, women with higher BP values show higher levels of MDA. Diminution of antioxidants may lead to vascular endothelial cells damage, and thus during preeclampsia total defensive ability of the plasma is not sufficient to overcome the oxidative stress and its consequences [23].

The immune system has also been shown to play a key role in the pathogenesis of AH; it has been shown to activate and migrate inflammatory cells, which accumulate in tissues to promote inflammation and initiate and deepen the remodeling of CV tissue [12]. Endothelial and VSMC stretching induced by an increase in intravascular pressure stimulates NADPH oxidase, leading to the production of ROS; activation of the RAS only enhances this effect. One of the RAS—angiotensin II (AT-II)—is a highly proinflammatory, vasoconstricting peptide which promotes oxidative stress in peripheral circulation [12,13]. In the pathophysiology of arterial hypertension, AT-II binds to angiotensin II type 1 (AT1) receptors, which leads to G-protein conjugation and causes signaling via secondary messengers, including diacylglycerol, which, as a strong endogenous activator of protein kinase C, contributes to the activation of phagocytic NADPH oxidase by p47phox [24]. As a consequence, oxidative cell damage and subsequent endothelial dysfunction occurs, leading to the development of hypertension. [12]. Furthermore, ROS-mediated oxidative stress contributes to increased signaling of the renin-angiotensin-aldosterone system (RAAS), favoring the development of CVD. Thus, it can be seen that the pathways of oxidative stress and inflammation interpenetrate, activating and intensifying each other’s effects. [25]. As a consequence, transcriptional proinflammatory factors, MAP kinases and profibrogenic mechanisms are activated. The development of vascular inflammation, which is mainly induced by the expression of proinflammatory genes modulated by transcription factors, contributes to arterial fibrosis and remodeling [17].

Increased age correlates to a higher incidence of CVD. AH is only one of many diseases that arises due to the vascular changes (e.g., stiffening of large elastic arteries and ED). These factors are caused by the loss of the balance between oxidants and antioxidants, as well as inflammation [6]. The developing vasoconstrictive effects, increased stiffness and vasculitis appear physiologically with increasing age, but AH accelerates this process [6,12]. The hope for restoring the proper functioning of the vessels is placed on antioxidants, and pharmacological strategies are still being sought to reduce oxidative stress and reverse the harmful effects of free radicals [12].

Age-related progressive ED may also affect skeletal muscle feed arteries (SMFAs); Park et al. [19] set out to investigate the role of mitochondrial free radicals in this disorder. The study used the mitochondria targeted antioxidant MitoQ to repair age-related ED. It has been observed that vasodilation in SMFA in response to flow and ACh is impaired in people with advanced age, and the use of MitoQ helps restore normal endothelial function by improving vasodilatation capacity. This study suggests that antioxidant supplementation may be effective in counteracting age-related vascular dysfunction [19]. Similar conclusions can be drawn from the meta-analysis conducted by Bredemeier and colleagues [26], who investigated the efficacy and role of xanthine oxidase inhibitors (XOI) in CVD, including AH. During purine metabolism, the ROS are produced in excess, leading to ED due to diminished production of NO. It is suggested that the use of the antioxidant features of XOI may reduce oxidative stress and decrease elevated BP. Moreover, by reducing the level of uric acid (UA), XOI also promotes an anti-inflammatory effect. While the suggested benefits of XOI on the reduction of oxidative stress and arterial hypertension may be a promising hypothesis, further research is needed to measure the biomarkers of oxidative stress [26].

## 4. Background Information on Oxidative Stress Biomarkers. Methods Used to Measure Oxidative Stress and Inflammation in the Studies Cited

Oxidative stress can be measured using appropriate biomarkers. They are important in assessing both pathological states and the positive effects of antioxidants on the body [27]. There are many types of biomarkers, with distinct applications [28]. Frijhof et al. [29] defined the characteristics that a biomarker should have in order to be clinically useful: disease specificity, prognostic value and correlation with disease activity [29].

There are several ways to examine the effects of specific agents on oxidative stress and inflammation. Most commonly, ROS-induced modifications are used to measure oxidative stress. For this purpose, oxidative stress biomarkers, such as advanced glycation end products (AGEs), oxidized low-density lipoprotein (oxLDL), lipid oxidation products, trans-4-hydroxy-2-nonenal (4-HNE), MDA, F_2_-isoprostanes (F_2_-IsoPs) or similar isolevuglandins (IsoLGs), are used, as they reflect the action of ROS in biological systems. This group also includes the stable inflammatory marker nitrotyrosine (Tyr-NO_2_), in addition to thiols, which are, however, dependent on many factors, and non-protein thiols, namely glutathione (GSH) and cysteine. Methionine sulfoxide, which is formed by ROS oxidizing the sulfur contained in the methionine molecule to sulfoxide, plays a role in the pathogenesis of pulmonary edema. Oxidized nucleosides are also used as biomarkers of total-body oxidative stress. We should also mention the markers of ROS generation, for example XO, which causes the conversion of xanthine to UA, which is not only an antioxidant but also a proinflammatory factor by influencing the NALP3 inflammasome. Myeloperoxidase (MPO) is an enzyme involved in both immune system reactions and in the pathogenesis of, among other disorders, atherosclerosis and other inflammatory CVD. Antioxidant defense is also an important factor, which we can assess using specific biomarkers, including protein thiol-disulfide oxidoreductases (such as thioredoxin (Trx) and peroxiredoxins (Prxs)) and nuclear factor (erythroid-derived 2)-like 2 (NRF2). Other ROS-related biomarkers include asymmetric dimethyl L-arginine (ADMA), whose elevated levels indicate the presence of CVD, and phosphorylated vasodilator-stimulated phosphoprotein (P-VASP), whose decreased levels are a sign of pathological cyclic guanosine monophosphate (cGMP) signaling [29].

For works cited in this article, biomarkers such as total antioxidant capacity (TAC), which was measured by colorimetric method, MDA [30,31], which was tested quantitatively by reaction with thiobarbituric acid reactive substance (TBARS) [30], and malondialdehyde-LDL (MDA-LDL) [32] were used (among others) to assess oxidative stress. Further, enzyme-linked immunosorbent assay (ELISA) was used to determine the levels of oxLDL [33], C-reactive protein (CRP) [34] and 8-isoprostanes (8-iso)/8-iso-prostaglandin F2α (8-iso-PGF2-α)/8-epi-prostaglandin F2α (8-epi-PGF2-α) [33,35,36,37,38]. ADMA levels were determined by either a standard curve of synthetic ADMA [37] or by the ELISA competitive method [33]. Nitrite and nitrate (NOx) were determined by the classic Griess method [38,39]. Luminex microbeads array system was used to measure matrix metalloproteinase-9 (MMP-9), MPO and adiponectin. To assess reactive oxygen metabolites (ROMs), the derivatives of reactive oxygen metabolites (d-ROMs) assay can be used. It involves the determination of serum levels of hydroperoxides, which react with a chromogenic substance to form a colored compound. The reaction is based on the Fenton reaction and is a photometric method—at a wavelength of 505 nm the amount of colored derivative produced is measured [40].

Interleukin-6 (IL-6) [30,33,41,42,43] immunoenzymatic method [33,41] was used to assess inflammation. Other inflammatory parameters determined in the same way were interleukin-1β (IL-1β) and interleukin-8 (IL-8) [43]. ELISA was used to detect intracellular adhesion molecule-1 (ICAM-1) [33,41,43], vascular endothelial adhesion molecule-1 (VCAM-1) [41,43] and TNF-α [35,43]. High-sensitivity C-reactive protein (hs-CRP) [41,42] was determined by an automated enzymatic test [41], a latex particle-enhanced immunoturbidimetric test [33] or by using anti-CRP monoclonal antibodies [40].

However, Marrocco et al. [27] emphasize that there are a number of mistakes that researchers make when assessing oxidative stress. These include lack of validation, standardization and reproducibility of measurement, for example, when determining the total antioxidant capacity of human body fluids or examining markers based on ROS-induced modifications of lipids, DNA and proteins [27]. Furthermore, Frijhof et al. [29] emphasize that researchers measure the activity of numerous biomarkers using many different, often non-specific, methods. As a result, sometimes these biomarkers do not adequately reflect the oxidative stress state and/or do not correlate with each other [29].

All of the oxidative stress and inflammation markers that were used in the studies cited in this article are listed in Table 2.

## 5. Effects of Diet and Supplementation on Oxidative Stress and Inflammation and Their Correlation with Blood Pressure 

Lifestyle changes, including dietary modification, are an important part of the management of AH. Following a healthy, balanced diet will delay or prevent the development of AH, while in hypertensive patients it will help lower BP [1]. There are many studies discussing the effects of specific nutrients on oxidative stress and inflammation and the relationship of the above with CVD. In this paper, we will focus on the effect of antioxidant and anti-inflammatory properties of nutrients on BP [30,31,34,35,41,44,45,46].

### 5.1. Antioxidant and Anti-Inflammatory Effects of Pomegranate Juice and Its Influence on Blood Pressure

Pomegranate juice has been shown to have antioxidant and anti-inflammatory properties due to its content of various types of antioxidants and polyphenols. As mentioned before, there is a strong correlation between oxidative stress and inflammation and BP. Consistent with these findings are studies on the effects of pomegranate juice consumption on, among other things, BP [30,41]. Pomegranate juice has been shown to have beneficial effects on both systolic and diastolic BP in end-stage renal disease (ESRD) patients on dialysis. BP significantly decreased in the group of patients consuming pomegranate juice (*p* < 0.001), in contrast to the control group where it significantly increased (*p* < 0.001) [30]. Similar findings came from a study by Asgary et al. [41] that examined the effect of pomegranate juice consumption on BP values in hypertensive patients. In contrast to the control group, the experimental group showed a decrease in SBP (*p* = 0.002) and DBP (*p* = 0.038), as well as a decrease in serum concentrations of the biomarkers for endothelial function and vascular inflammation (VCAM-1) (*p* = 0.008) [41]. It is worth noting that the study by Barati Boldaji et al. [30] had a greater number of participants than the study by Asgary et al. [41] (41 experimental group/40 control group vs. 21 participants. It is also remarkable that other biomarkers of oxidative stress and inflammation were measured in the studies (Table 2). Furthermore, the study by Barati Boldaji et al. [30], unlike the trial by Asgary et al. [41], included ESRD patients on dialysis treatment, which may have influenced the outcome of the study. Despite this, in both studies the decrease in SBP and DBP was correlated with the antioxidant and anti-inflammatory effects of pomegranate juice [30,41]. However, Asgary et al. [41] points out that further double-blind studies are needed. In addition, geographical region may affect the phytochemical composition of pomegranate, which may affect its properties [30]. The results of both studies comparing the change in blood pressure in the study group and the control group are shown in Table 3 and Table 4.

### 5.2. Impact of Daily Blueberry or Strawberry Consumption on Blood Pressure in Pre- and Stage 1-Hypertensive Postmenopausal Women

It is commonly understood that postmenopausal women are particularly vulnerable to the development of chronic diseases. The reason is estrogen deficiency, which predisposes to increased oxidative stress and chronic inflammation [35]. It is also worth noting that in patients with pre- and stage 1-hypertension, the primarily recommendation is lifestyle and dietary changes [44]. Hence the hypotensive effect of daily consumption of blueberries and strawberries on a group of pre- and stage 1-hypertensive, postmenopausal women aged 45–65 years was evaluated [34,35,44]. This was successful with blueberries, where significant decreases in SBP (*p* < 0.05) and DBP (*p* < 0.01) were observed with no significant decreases in the control group [34,35]. In contrast, daily consumption of strawberries resulted only in a significant decrease in SBP (−6 mmHg) in the group consuming 25 g freeze-dried strawberry powder (FDSP). However, no other statistically significant changes in BP were observed. Nevertheless, Feresin et al. [44] emphasize that these results are clinically relevant because study participants in both experimental groups (consuming 25 and 50 g of FDSP) regressed from stage 1 hypertension at the beginning of the study to a prehypertensive state at the end of the study [44].

Similar to pomegranate juice (thanks to its content of, among other things, dietary fiber and polyphenols (in particular flavonoids)), blueberries and strawberries have antioxidant and anti-inflammatory effects, thus protecting against the development of chronic diseases such as AH [34,35,44]. Despite this, the cited studies did not show antioxidant and anti-inflammatory effects of blueberries and strawberries [35,44]. Daily consumption of blueberries, contrary to expectations, did not result in improvements in either oxidative DNA damage or in circulating biomarkers [35]. Daily consumption of strawberries did not result in the expected increase in the activity of the major antioxidant enzyme SOD [44]. The authors of both studies indicate that additional research is needed [35,44].

### 5.3. Effects of Chia Supplementation on Blood Pressure and Its Antioxidant and Anti-Inflammatory Properties

The antioxidant and anti-inflammatory effects of nutrients and their effects on BP have also been studied with chia supplementation (*Salvia hispanica* L.) [31,45,46]. Toscano et al. [31] studied treated and untreated hypertensive patients. The study participants were divided into 3 groups: chia group previously treated with medication (CHIA-MD), chia group without medication (CHIA-NM) and placebo group with medication (PLA-MD). Medications used in the hypertension treatment groups included diuretics, CCBs, ACE-I and AT1 receptor blockers. A decrease in both SBP and DBP was observed in the CHIA and CHIA-MD groups, resulting in a significantly decreased mean blood pressure (MBP). In contrast, a significant decrease in only SBP was observed in the CHIA-NM group, with a nonsignificant decrease in MBP values. The results were confirmed by ABPM. It has also been shown that chia supplementation can lower BP in patients resistant to pharmacological treatment [31]. Similarly, a study by Alwosais et al. [45] showed a reduction in SBP of about 13 (±10) mmHg over the course of the study [45]. In the study by Toscano et al. [31], it is noteworthy that each of the patient groups was composed of only 7 to 10 participants; Alwosais et al. [45] also indicate the need for further studies with larger sample sizes.

The relationship between chia’s antioxidant and anti-inflammatory properties to its hypotensive effects remains unclear, with a demonstrated reduction in lipid peroxidation accompanied by no change in inflammatory markers; however, the authors of this study suggest that additional studies are needed [31]. Such a study was undertaken by Orona-Tamayo et al. [46], who demonstrated that chia seeds possess free radical scavenging, ferrous ion chelating and ACE-I properties. Thus, they possess antihypertensive potential due to their antioxidant properties [46].

In conclusion, the results show that the consumption of chia is beneficial and is able to produce hypotensive effects in hypertensive patients [31,46].

Most of the studies cited indicate that the addition of a single dietary ingredient can have a beneficial effect on BP values [30,31,34,35,41,44,45]. Although the antioxidant and anti-inflammatory properties of specific nutrients are known, their association with hypotensive effects is not clear. Furthermore, the study authors suggest that additional research is needed on this topic [31,35,44]. The effects of the aforementioned nutrients on oxidative stress and inflammation are shown in Table 4, while Table 5 and Table 6 show how they affected SBP and DBP values, respectively.

## 6. Pharmacological Therapy of Arterial Hypertension and Its Effects on Oxidative Stress and Inflammation

In many patients, lifestyle modification may not be sufficient to achieve optimal BP values. It is then necessary to implement pharmacological treatment of AH. Pharmacological therapy is based on five classes of drugs and their combinations: ACE-I, ARBs, beta-blockers, CCBs, thiazides and thiazide-like diuretics. In hypertensive patients for whom the main classes of drugs are not effective, groups of drugs, such as alpha-blockers, centrally acting agents and mineralocorticoid receptor antagonists, may complement the therapy [1].

### 6.1. Characterization of ACE-I and ARBs; Antioxidant and Anti-Inflammatory Properties of Selected Examples

ACE-I and ARBs are the most commonly used [1] groups of hypotensive drugs; they have similar efficacy and have been studied in many clinical trials [1,4]. They reduce the risk of atrial fibrillation (AF) and are indicated in patients with chronic heart failure with reduced ejection fraction (HFrEF) and those with a history of myocardial infarction (MI). They also reduce CV incident rates and patient mortality. It is worth noting that ACE-I and ARBs delay the progression of chronic kidney disease (CKD), including diabetic nephropathy, and reduce albuminuria. In contrast, it has been observed that the use of ACE-I or ARBs results in an increase in creatinine concentration and causes a functional reduction in estimated glomerular filtration rate (eGFR) due to the reduction in BP. Therefore their concomitant use is not recommended due to an increased risk of renal events and no benefit from such a drug combination. Although both groups of drugs are characterized by similar side effects, ARBs are less likely to cause cough and angioedema than ACE-I, and far fewer patients need to discontinue therapy due to side effects [1,4].

ACE-I limit the conversion of angiotensin I to AT-II. Due to the presence of other enzymes involved in AT-II synthesis, the use of ACE-I does not fully inhibit the formation of AT-II, but only reduces its level [47]. ARBs work by blocking the binding of AT-II to its high-affinity AT1 receptor [48]. RAS blockade reduces the biological activity of AT-II [47,48], which plays an important role in oxidative stress and inflammation in CVD, specifically, it starts an inflammatory cascade involving NADPH oxidase, ROS and inflammatory nuclear transcription factor [40].

**Figure 2:**
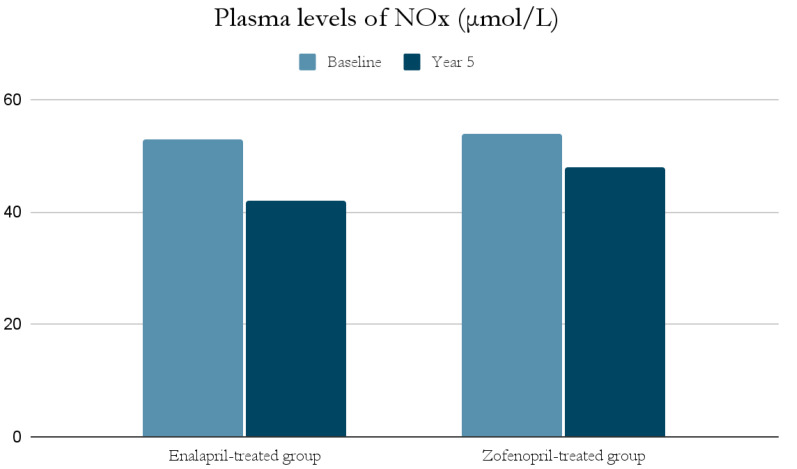
Comparison of plasma levels of NOx (μmol/L) at baseline and at year 5 in the enalapril and zofenopril groups. Newly diagnosed hypertensive patients (SBP > 160 mm Hg and/or DBP > 95 mm Hg) participated in the study. They were randomly assigned to receive enalapril (20 mg/d, n = 24) or zofenopril (30 mg/d, n = 24). Exclusion factors were additional risk factors for coronary artery disease or a history of ischemic events, as well as prior or concurrent therapy with ACE-I, antiplatelet agents or anticoagulants [37].

**Figure 3:**
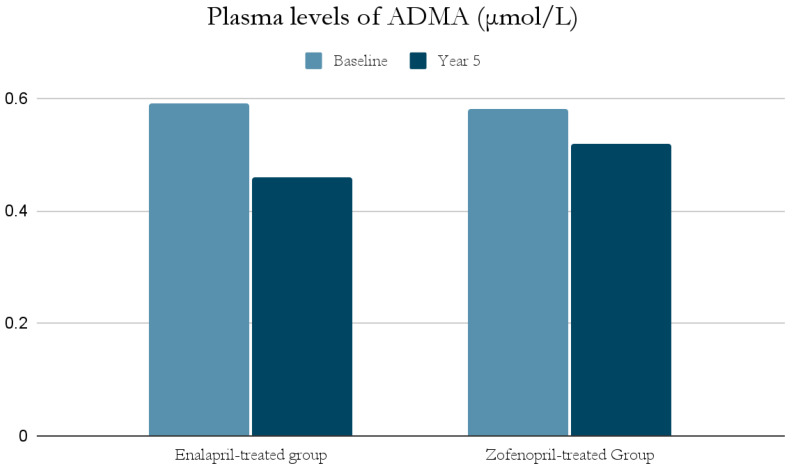
Comparison of plasma levels of ADMA (μmol/L) at baseline and at year 5 in the enalapril and zofenopril groups. Newly diagnosed hypertensive patients (SBP > 160 mm Hg and/or DBP > 95 mm Hg) participated in the study. They were randomly assigned to receive enalapril (20 mg/d, n = 24) or zofenopril (30 mg/d, n = 24). Exclusion factors were additional risk factors for coronary artery disease or a history of ischemic events, as well as prior or concurrent therapy with ACE-I, antiplatelet agents or anticoagulants [37].

The ACE-I group includes drugs that differ in their functional group, which can alter the effect of a particular drug. For example, enalapril has a carboxylate group and zofenopril has two sulfhydryl groups. It has been observed that both enalapril and zofenopril treatment resulted in a significant reduction in plasma NOx and ADMA concentrations (Figure 2 and Figure 3) [37].

The decrease in NOx levels may be due to the fact that abnormal NO-dependent vascular relaxation is observed in hypertensive subjects [37,39]. This may be related to the presence of inducible nitric oxide synthase (iNOS) and increased expression of proinflammatory genes [49]; as mentioned previously, inflammatory cell infiltration and developing inflammation in the vessel wall have a significant role in the pathogenesis of AH. This is also confirmed by studies on animals suggesting an association of AH with low-grade inflammation [49]. In addition, both NOx and ADMA levels in hypertensive patients were significantly higher at the beginning of the study than in normotensive subjects, and decreased significantly after blood pressure was lowered by ACE-I treatment [37,39].

Moreover, the antioxidant properties of both drugs were demonstrated by lowering the isoprostane 8-iso-PGF2α concentration. However, its reduction was greater in the zofenopril group. This phenomenon may be explained by the presence of sulfhydryl groups, which are capable of scavenging free radicals and therefore may correspond to the antioxidant capacity of zofenopril. Nevertheless, the study authors emphasize that there is a risk of a dose-dependent effect due to a higher dose of zofenopril than enalapril being administered [37,38].

Antioxidant and anti-inflammatory properties have been also demonstrated with irbesartan (Table 7) [32,40], which is an ARB and acts as a partial agonist of PPAR-γ [32,40,50]. A change in treatment from another ARB to irbesartan resulted in a decrease in hs-CRP (2.80 ± 0.53 versus 2.66 ± 0.50 log(ng mL^−1^)) and d-ROMs (338 ± 74 versus 305 ± 62 U.CARR; *p* < 0.001); there was also a decreasing trend in the proteolytic enzyme released from inflammatory cells, MMP-9 (1.03 ± 0.86 versus 0.62 ± 0.59 mg mL^−1^; *p* = 0.062) and MPO (1.50 ± 0.70 versus 0.51 ± 0.43 IU mL^−1^; *p* = 0.057), which plays a role in oxidative and inflammatory processes. The change in d-ROM levels correlated positively with the change in hs-CRP levels. Moreover, the antioxidant effect of irbesartan is independent of its dose, but may be related to its anti-inflammatory effect [40]. On the contrary, a study by Umebayashi et al. [32] found no effect of changing ARB to irbesartan on markers of oxidative stress and inflammation. However, the authors suggest that this is due to the small number of high-risk patients (≤10%) and thus the mean hs-CRP value characterizing the patients was too low [32]. Umebayashi et al. [32] also point out that the comparison of irbesartan with other ARBs in in vitro studies provides different conclusions. In these studies, irbesartan was more potent than losartan in reducing the production of inflammatory cytokines, such as monocyte chemoattractant protein-1 (MCP-1) [50]. The studies by Taguchi et al. [40] and Umebayashi et al. [32] cited above are summarized in Table 7, which presents a number of differences that characterize the two studies, such as study design, number of patients and their characteristics and duration of therapy. The juxtaposition of the two studies is intended to compare the results of the analysis of the antioxidant and anti-inflammatory properties of irbesartan, and the values presented in Table 7 may influence the different results. Most noteworthy is the significantly longer duration of the Umebayashi et al. [32] study compared to the Taguchi et al. [40] trial (6 months vs. 12 weeks). Furthermore, the Taguchi et al. [40] trial involved 118 patients, whereas the Umebayashi et al. [32] trial involved 76 patients. 

The anti-inflammatory properties of candesartan have been described in a study by Derosa et al. [42]. The experiment studied the effect of this drug on inflammation associated with postprandial hyperlipidemia (induced by oral fat load (OFL)) in type 2 diabetic and non-diabetic hypertensive patients. Candesartan therapy significantly affected soluble intercellular adhesion molecule-1 (sICAM-1) (−17.3%), IL-6 (−31.6%) and Hs-CRP (−33.3%) levels in non-diabetic subjects and sICAM-1 (−16.5%), IL-6 (−36.6%) and Hs-CRP (−25.0%) in diabetic subjects. Moreover, it caused a smaller OFL-related increase in IL-6 (+28.6 vs. +47.4%) and Hs-CRP (+62.5 vs. +75.0%) in non-diabetic patients and in sICAM-1 (+6.5 vs. +8.5%) and IL-6 (+36.4 vs. +48.5%) in diabetic patients. Candesartan therapy attenuates the inflammatory response in patients both with and without diabetes; however, the anti-inflammatory effect of candesartan is more significant in non-diabetic patients. The authors point to the disadvantages of a relatively small number of participants and the fact that only the most common inflammatory biomarkers were measured [42].

### 6.2. Characteristics of Beta-Blockers and Antioxidant and Anti-Inflammatory Properties Using Metoprolol and Nebivolol as Examples

Beta-blockers are indicated for the treatment of AH in patients with angina pectoris, for the control of heart rate after MI and in patients with HFrEF. They are an alternative to ACE-I and ARBs in women planning pregnancy and of reproductive age. Beta-blockers have been proven to reduce the risk of both HF and stroke, as well as other major CV events. Major drawbacks with beta-blocker therapy, especially when combined with diuretics, is an increased risk of developing diabetes, and their combination with verapamil or diltiazem causes a serious risk of bradycardia. They are characterized by more side effects than RAS blockers, resulting in more patients having to discontinue therapy [1,4]. The antioxidant and anti-inflammatory properties of metoprolol and nebivolol were investigated. It was shown that both metoprolol and nebivolol significantly reduced oxLDL levels (*p* < 0.01) and ICAM-1 levels (*p* < 0.01) in hypertensive patients. In contrast, nebivolol alone appeared to reduce 8-iso levels (*p* = 0.01). The reduction in oxLDL and 8-iso levels in nebivolol-treated patients was independent of the reduction in BP, in contrast to metoprolol, whose effect on oxLDL levels was associated with a change in BP. It is noteworthy that no changes were observed in the concentrations of inflammatory markers (hsCRP, white blood cells (WBC), fibrinogen and IL-6) and ADMA. Serg et al. [33] emphasize that nebivolol appears to be preferable to metoprolol in terms of systemic antioxidant properties. The disadvantages of the study include the relatively small number of participants and the fact that 12 patients in the nebivolol group and 9 patients in the metoprolol group began taking hydrochlorothiazide concomitantly after week 4 of the study, which may have affected oxidative stress [33].

### 6.3. Characteristics of Calcium Channel Blockers and Thiazides/Thiazide-like Diuretics; Comparison of Their Antioxidant and Anti-Inflammatory Properties

CCBs are effective antihypertensive drugs, whose effects on CV risk and mortality are comparable to other classes of hypotensive drugs. They strongly reduce stroke risk, but are less effective in preventing HFrEF. A common side effect of CCBs is the occurrence of peripheral edema; however, the incidence of peripheral edema has been shown to be 38% lower with CCB + ACE-I/ARB therapy. It is worth noting that CCBs inhibit the cytochrome P450 3A4 enzyme, and thus may cause interactions with other drugs [1,4].

Thiazide and thiazide-like diuretics are antihypertensive drugs effective in preventing CV incidents and CV mortality [1]. Their common side effect is electrolyte disorders, such as hyponatremia and hypokalemia, so combining them with potassium-sparing hypotensive drugs, such as ACE-I, ARBs or potassium-sparing diuretic, may be beneficial [4]. Their effect is reduced in patients with eGFR < 45 mL/min and completely stops at eGFR < 30 mL/min, which is explained by their mechanism of action [1].

A comparison of the effects of ARB + CCB (olmesartan + amlodipine) and ARB + thiazide diuretics (olmesartan + hydrochlorothiazide) therapy showed that both drug combinations resulted in a decrease in CRP. However, only ARB + CCB combination therapy (olmesartan + amlodipine) caused a decrease in all tested inflammatory factors: TNF-α (16.1% decrease), IL-1β (18.5%), IL-6 (18.1%), IL-8 (12.8%), ICAM-1 (20.8%) and VCAM-1 (30.8%). Thus, antihypertensive therapy containing a combination of ARB and CCB is more beneficial with regards to inflammatory processes than therapy based on an ARB and thiazide diuretic [43]. The results of the study by Zhou et al. [51] seem consistent. They revealed that both thiazide diuretics (hydrochlorothiazide) and thiazide-like directives (chlorthalidone) have no effect on oxidative stress—they did not reduce the expression of lectin-like oxidized low density lipoprotein receptor-1 (LOX-1), MCP-1 and AT1 receptor molecules, nor did they prevent an increase in ROS production [51].

Most of the antioxidant and/or anti-inflammatory properties of the major groups of hypotensive drugs have been revealed [33,37,38,40,42,43]. The aforementioned studies are summarized in Table 8. Antihypertensive treatment ameliorates the increased oxidative stress that is present in hypertensive patients. It is well known that ROS products are involved in the development of ED and organ damage. It therefore follows that drug therapy for AH may help prevent organ damage [52].

**Figure 4:**
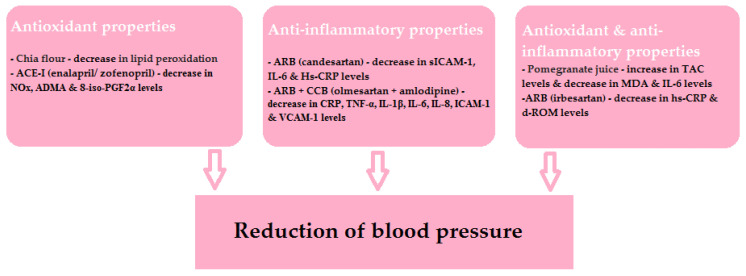
The beneficial effects of the discussed drugs and specific nutrients.

The beneficial effects of the drugs and specific nutrients described in this article are shown schematically in Figure 4. It is worth remembering that, as mentioned above, the relationship of the antioxidant and anti-inflammatory properties of individual nutrients and their hypotensive effects is not clear. However, it is evident that ROS products are involved in the development of ED and organ damage.

## 7. Conclusions

In our publication we focused on the correlation of AH with oxidative stress and inflammation, which are influenced by many factors, such as diet, supplementation and drug treatment. It is believed that the imbalance between the amount of oxidants and antioxidants production results in DNA, protein and lipid damage. By contributing to cell and tissue impairment, these processes promote the development and progression of AH. The underlying causes of this multifactorial disease are numerous processes, among which ED, inflammation, increased vascular contractility and remodeling play crucial roles in inducing stiffening of large elastic arteries, loss of stiffness, other changes to mechanical properties and remodeling of CV tissue. Studies show that the addition of a single dietary ingredient can have a beneficial effect on BP values. Although the association of the hypotensive effect with the antioxidant and anti-inflammatory properties of blueberries, strawberries and chia requires further study, it was revealed that there is a correlation between the decrease in SBP and DBP and the antioxidant and anti-inflammatory effects of pomegranate juice. The effect of drug treatment of AH on oxidative stress and inflammation has also been analyzed. The study shows that most of the major groups of hypotensive drugs have antioxidant and/or anti-inflammatory properties. Thus, the use of drug therapy in hypertensive patients seems to provide protection against organ damage.

## Figures and Tables

**Table 1:** Most common ROS divided into oxygen-free radicals and non-radical ROS.

Oxygen-Free Radicals	Non-Radical ROS
Superoxide	Hydrogen peroxide
Hydroxyl radical	Peroxynitrite
Peroxyl radical	Hypochlorous acid
Alkoxy radical	Ozone

ROS, reactive oxygen species.

**Table 2:** Biomarkers that have been tested to determine the antioxidant and/or anti-inflammatory properties of a substance/drug.

Study Author	Biomarkers of Oxidative Stress or Inflammation
Barati Boldaji et al. [30]	TAC, MDA, IL-6
Toscano et al. [31]	hs-CRP, AGP, MDA, Nitrite
Umebayashi et al. [32]	hsCRP, PTX3, MCP-1, MDA-LDL
Serg et al. [33]	OxLDL, 8-iso *, ICAM-1, ADMA, IL-6, hsCRP, Fibrinogen, WBC
Johnson et al. [34,35]	CRP, GPx, GR, 8-OHdG, 8-iso *, OxLDL, SOD, TBARS, TNF-α
Napoli et al. [36,37,39]	NOx, 8-iso-PGF2-α */8-epi-PGF2-α *, ADMA
Cacciatore et al. [38]	NOx, 8-iso-PGF2-α *
Taguchi et al. [40]	hs-CRP, MMP-9, d-ROMs, MPO, Adiponectin
Asgary et al. [41]	ICAM-1, VCAM-1, hs-CRP, IL-6
Derosa et al. [42]	sICAM-1, IL-6, hs-CRP
Martinez-Martin et al. [43]	TNF-α, CRP, IL-1β, IL-6 and IL-8, ICAM-1, VCAM-1
Feresin et al. [44]	SOD

TAC, total antioxidant capacity; MDA, malondialdehyde; IL-6, interleukin-6; ICAM-1, intracellular adhesion molecule-1; VCAM-1, vascular endothelial adhesion molecule-1; hs-CRP, high-sensitivity C-reactive protein; CRP, C-reactive protein; GPx, glutathione peroxidase; GR, glutathione reductase; 8-OHdG, 8-hydroxy-2′-deoxyguanosine; 8-iso, 8-isoprostane; OxLDL, oxidized low-density lipoprotein; SOD, superoxide dismutase; TBARS, thiobarbituric acid reactive substances; TNF-α, tumor necrosis factor-α; AGP, α-1 acid glycoprotein; MMP-9, matrix metalloproteinase-9; d-ROMs, derivatives of reactive oxygen metabolites; MPO, myeloperoxidase; NOx, nitrite and nitrate; 8-iso-PGF2-α, 8-iso-prostaglandin F2α; 8-epi-PGF2-α, 8-epi prostaglandin F2α; ADMA, asymmetric dimethylarginine; PTX3, pentraxin3; MCP-1, monocyte chemoattractant protein-1; MDA-LDL, malondialdehyde-LDL; sICAM-1, soluble intercellular adhesion molecule-1; WBC, white blood cells; IL-1β, interleukin-1β; IL-8, interleukin-8. * The authors of this studies used the names 8-iso [33,34,35] or 8-iso-PGF2-α/8-epi-PGF2-α [36,37,38], however, all reported that they used the 8-isoprostane ELISA kit (Cayman Chemical Company^®^, Ann Arbor, MI, USA), which does not distinguish between these compounds, so they should not be understood as separate compounds in the table presented.

**Table 3:** The difference in SBP (mm Hg) and DBP (mm Hg) between pre-test and post-test values.

Authors	Barati Boldaji et al. [30]	Asgary et al. [41]
Patient category	ESRD patients on dialysis treatment, aged 18–65 years with serum potassium level of less than 6 mEq/L.	Hypertensive patients SBP > 140 mmHg and/or DBP > 90 mmHg) aged 30–67 years with BMI ≤ 30.
Difference in SBP (mm Hg) between pre-test and post-test values		
Experimental group	has decreased	has decreased
Control group	has increased	-
*p*-value	<0.001	0.002
Difference in DBP (mm Hg) between pre-test and post-test values		
Experimental group	has decreased	has decreased
Control group	has increased	-
*p*-value	<0.001	0.038

ESRD, end-stage renal disease; SBP, systolic blood pressure; DBP, diastolic blood pressure; BMI, body mass index.

**Table 4:** Effects of specific nutrients on oxidative stress and inflammation.

Authors	Barati Boldaji et al. [30]	Toscano et al. [31]	Johnson et al. [34,35]	Asgary et al. [41]	Feresin et al. [44]
Study design	Randomized Controlled Trial	Randomized Controlled Trial	Randomized Controlled Trial	Clinical Trial	Randomized Controlled Trial
All patients	41 experimental group/40 control group	26	40	21	60
Patient category	ESRD patients on dialysis treatment, aged 18–65 years with serum potassium level of less than 6 mEq/L.	Hypertensive patients (mild/stage 1 hypertension according to the VI Brazilian Guidelines on Hypertension) aged 35–65 years with BMI between 25 and 35 kg/m^2^.	Pre- and stage 1-hypertensive postmenopausal women aged 45–65 years.	Hypertensive patients (SBP > 140 mmHg and/or DBP > 90 mmHg) aged 30–67 years with BMI ≤ 30.	Pre- and stage 1-hypertensive postmenopausal women aged 45–65 years.
Type of product	Pomegranate juice	Chia flour	Freeze-dried blueberry powder	Pomegranate juice	FDSP
Antioxidant/Anti-inflammatory effects	Antioxidant and anti-inflammatory properties have been demonstrated by increasing TAC levels and decreasing MDA and IL-6 levels.	A reduction in lipid peroxidation with no change in inflammatory markers was demonstrated.	Antioxidant and anti-inflammatory effects were not demonstrated, as no improvement in oxidative DNA damage and circulating biomarkers was observed at the end of the study.	Anti-inflammatory properties were demonstrated by significant reduction in the levels of the endothelial function and vascular inflammation biomarker VCAM-1.	Antioxidant and anti-inflammatory effects were not demonstrated because there was no increase in SOD activity.

FDSP, Freeze-dried strawberry powder.

**Table 5:** Effects of specific nutrients on SBP values.

Authors	Toscano et al. [31]	Johnson et al. [34,35]	Feresin et al. [44]
Type of product	Chia flour	Freeze-dried blueberry powder	FDSP
SBP (mm Hg)			
(1) Baseline			
Experimental group	146.2 ± 2.0 (CHIA) 145.8 ± 2.2 (CHIA-MD) 146.8 ± 3.8 (CHIA-NM)	138 ± 14	141 ± 3 (25 g FDSP group) 142 ± 3 (50 g FDSP group)
Control group	144.0 ± 4.3	138 ± 15	137 ± 3
(2) Post-trial			
Experimental group	136.3 ± 2.6 (CHIA) 133.7 ± 4.1 (CHIA-MD) 137.3 ± 3.1 (CHIA-NM)	131 ± 17	135 ± 3 (25 g FDSP group) 138 ± 3 (50 g FDSP group)
Control group	141.2 ± 5.2	139 ± 15	132 ± 3

**Table 6:** Effects of specific nutrients on DBP values.

Authors	Toscano et al. [31]	Johnson et al. [34,35]	Feresin et al. [44]
Type of product	Chia flour	Freeze-dried blueberry powder	FDSP
DBP (mm Hg)			
(1) Baseline			
Experimental group	94.2 ± 2.0 (CHIA) 94.3 ± 2.4 (CHIA-MD) 94.2 ± 3.6 (CHIA-NM)	80 ± 7	81 ± 2 (25 g FDSP group) 79 ± 2 (50 g FDSP group)
Control group	90.1 ± 2.4	78 ± 8	79 ± 2
(2) Post-trial			
Experimental group	85.5 ± 1.2 (CHIA) 83.3 ± 1.3 (CHIA-MD) 88.7 ± 1.8 (CHIA-NM)	75 ± 9	79 ± 2 (25 g FDSP group) 79 ± 2 (50 g FDSP group)
Control group	87.8 ± 2.2	80 ± 8	79 ± 2

**Table 7:** Comparison of studies examining the antioxidant and anti-inflammatory properties of irbesartan.

Authors	Umebayashi et al. [32]	Taguchi et al. [40]
Study design	Randomized controlled trial; Patients who had been taking ARBs except irbesartan for more than 3 months were divided into 2 groups, one continuing the same ARB and the other switching ARBs to irbesartan for 6 months.	Clinical trial; High-risk patients who were taking ARBs, except irbesartan, for over 3 months and had stable BP underwent 4-week follow-up, and then all ARBs were switched to an equivalent dose of irbesartan for 12 weeks.
All patients	76	118
Patient category	Hypertensive patients, aged 20–85, who had failed to achieve target BP levels (140/90 mmHg or 130/80 mmHg for patients with diabetes, chronic kidney disease or myocardial infarction) with conventional ARBs (losartan, candesartan, valsartan, olmesartan or telmisartan) for more than 3 months.	High-risk hypertensive patients with the presence of at least one complication, such as coronary artery disease, cerebrovascular disease, or diabetes.
Duration of irbesartan therapy	6 months	12 weeks
Antioxidant/Anti-inflammatory effects	There was no effect of changing ARB to irbesartan on markers of oxidative stress and inflammation.	The antioxidant and anti-inflammatory properties of irbesartan (decrease in hs-CRP and d-ROM) have been demonstrated.

ARB, angiotensin receptor blocker; BP, blood pressure.

**Table 8:** Comparison of antioxidant and anti-inflammatory properties of hypotensive drugs.

Authors	Serg et al. [33]	Napoli et al. [37]	Cacciatore et al. [38]	Taguchi et al. [40]	Derosa et al. [42]	Martinez-Martin et al. [43]
Study design	Randomized Controlled Trial	Prospective randomized clinical trial	Randomized Controlled Trial	Clinical trial	Controlled Clinical Trial	Randomized Controlled Trial
All patients	63	48	36	118	219	120
Patient category	Hypertensive patients (never-treated mild-to-moderate essential hypertension), aged 30–65 years.	Newly diagnosed patients with mild hypertension; without additional risk factors for atherosclerosis.	Newly diagnosed patients with mild hypertension; without CVD and associated risk factors, who are not receiving ACE-I therapy.	High-risk hypertensive patients with the presence of at least one complication, such as coronary artery disease, cerebrovascular disease, or diabetes.	Hypertensive non-diabetic (n = 106) and diabetic (n = 113) patients aged ≥18 years.	Hypertensive patients (stage I and II hypertension; SBP 140–179 mmHg) aged 25–75 years with MetS, as defined by the International Diabetes Federation for Europid populations.
Drug class	Beta-blockers (nebivolol/metoprolol)	ACE-I (enalapril/zofenopril)	ACE-I (enalapril/ zofenopril)	ARB (irbesartan)	ARB (candesartan)	ARB + CCB (olmesartan + amlodipine)/ARB + thiazide diuretics (olmesartan + hydrochlorothiazide)
Antioxidant/Anti-inflammatory effects	Decreased levels of oxLDL, ICAM-1, and 8-iso (nebivolol only) were observed, whereas there were no changes in inflammatory markers (hsCRP, WBC, fibrinogen, and IL-6) and ADMA.	There were reductions in plasma NOx and ADMA (more prominent in the enalapril group) and 8-iso-PGF2α (more prominent in the zofenopril group).	There was a plasma decrease in NOx (with no clear differences between treatment groups) and 8-iso-PGF2α (more prominent in the zofenopril group).	The antioxidant and anti-inflammatory properties of irbesartan (decrease in hs-CRP and d-ROM) have been demonstrated.	The anti-inflammatory properties were observed through its beneficial effects on inflammatory markers, such as sICAM-1, IL-6 and Hs-CRP.	Better anti-inflammatory properties of ARB + CCB combination than ARB + thiazide diuretics (decrease in CRP in both groups; decrease in TNF-α, IL-1β, IL-6, IL-8, ICAM-1, and VCAM-1 levels in ARB + CCB group only).

CVD, cardiovascular disease; MetS, metabolic syndrome; ACE-I, angiotensin-converting-enzyme inhibitor; CCB, calcium channel blocker.

## Data Availability

The data is contained within the article.
